# Multi-antibiotic resistant extended-spectrum beta-lactamase producing bacteria pose a challenge to the effective treatment of wound and skin infections

**DOI:** 10.11604/pamj.2017.27.66.10226

**Published:** 2017-05-30

**Authors:** Angus Nnamdi Oli, Dennis Emeka Eze, Thaddeus Harrison Gugu, Ifeanyi Ezeobi, Ukamaka Nwakaku Maduagwu, Chibueze Peter Ihekwereme

**Affiliations:** 1 Department of Pharmaceutical Microbiology and Biotechnology, Faculty of Pharmaceutical Sciences, Nnamdi Azikiwe University, Awka, Anambra State, Nigeria; 2 Department of Orthopedic Surgery, Chukwuemeka Odumegwu Ojukwu University Teaching Hospital Amaku-Awka, Anambra State, Nigeria; 3 Department of Pharmacology and Toxicology, Faculty of Pharmaceutical Sciences, Nnamdi Azikiwe University, Awka, Anambra State, Nigeria

**Keywords:** *E. coli*, SBL, bacteria isolates, multi-antibiotic resistance, wound/skin infections

## Abstract

**Introduction:**

The increasing incidence of antibiotic resistant bacteria is a concern both to the clinicians and the patients due to obvious consequences such as treatment failures, prolonged patients’ stay in hospital and nosocomial infections. The choice of the first antibiotic therapy in emergency wards in hospitals is usually not based on patient-specific microbial culture and susceptibility test result.This study is aimed at profiling extended-spectrum beta-lactamase (ESBL) producing bacteria associated with wound injuries and highlighting their multi-antibiotic resistance character.

**Methods:**

Sixty-three wound swab samples were collected and cultured on nutrient agar and on selective media. Evaluation for ESBL production was by phenotypic method while the antibiogram screening was by disc-diffusion.

**Results:**

The wounds evaluated were diabetic sore (14), cancer wounds (12), surgical wounds (17), wounds due to road traffic accidents (10) and wounds from fire burn (10). The result showed that 61 wounds were infected and the prevalence of the infecting pathogens was *Escherichia coli* 17.46%, *Klebsiella Pneumonia* 14.28%, *Salmonella typhi* 12.79%, *Pseudomonas Aeruginosa* 34.92% and *Staphylococcus aureus* 17.46%. Thirty four (55.74 %) isolates were ESBL producers, greater than 50% of which being *Pseudomonas Aeruginosa*. The antibiogram study of the ESBL producers showed multi-drug resistance with resistance highest against ampicillin (100%), followed by cephalosporins: cefuroxime (94.12%) and ceftriaxone (61.76%). No resistance was recorded against the β-lactamase inhibitors: amoxicillin/clavulanate and ceftriaxone/sulbactam. There was a high incidence (55.74 %) of ESBL-producing microbes in the wounds. The isolates were mostly multi-antibiotic resistant.

**Conclusion:**

Multi-drug resistant ESBL-producing bacteria are common in wound infections in the community. However, amoxicillin/clavulanate or ceftriaxone/sulbactam may be used to treat most patients with such infections in the hospital. This may guide antibiotic selection and use in trauma, most especially in resource limited countries where laboratory test is unaffordable for a majority of patients.

## Introduction

The increasing incidence of antibiotic resistant bacteria is a concern both to the clinicians and the patients due to obvious consequences such as treatment failures, prolonged patients’ stay in hospital and nosocomial infections. The choice of the first antibiotic therapy in emergency wards in hospitals is usually not based on patient-specific microbial culture and susceptibility test result. Extended spectrum β-lactamases (ESBLs) have been reported to be common cause of hospital-acquired infections and can have severe clinical implications with a corresponding multiple antibiotic resistances [1, 2]. The enzymes, β-lactamases, deactivate the molecular antibacterial properties of β-lactam antibiotics by breaking and opening the β-lactam ring. Bacteria harboring these enzymes are usually resistant to β-lactam antibiotics as well as antibiotics from other classes thereby posing a therapeutic challenge to clinicians [3]. ESBLs enzymes are carried in and transferred by bacteria plasmid [4, 5] and are responsible for bacteria resistance to β-lactam antibiotics but are inhibited by β-lactamase inhibitors [6]. Increase in ESBL-producing enteric Gram-negative bacteria has led to the choice of inappropriate therapy and consequently, the rate of resistance has increased. Antibiotic selection for infections due to ESBL-producing pathogens is still a clinical challenge. In most cases, carbapenems and fluoroquinolones have been used [7].

Horizontal gene transfer by plasmid exchange between *E. coli* strains is a recognized source of rapid spread of antimicrobial resistance phenotypes [4]. The understanding that plasmids are linked with mobile genetic elements highly suggests that they are responsible for transferring resistant genes to susceptible bacteria recipients [8]. Resistance to different classes of antibiotics had been detected among ESBL producers [9, 10]. Selective pressure of the antimicrobials selects those strains that are resistant to the applied antimicrobials causing the resistant strains to multiply and spread [11, 12].

Indiscriminate use of antibiotics, poor hygienic practices in hospitals and lack of monitoring for microbial drug resistance create suitable conditions for the emergency and spread of the ESBLs [10]. Other factors reported as fueling the spread of ESBLs in developing economies include extensive self-medication/prescribing and non-prescription use of antimicrobials, poor hygienic conditions even in the hospital environment and very low infection control practice [13, 14]. Knowledge on local antimicrobial resistance trends among wound isolates is important not only in guiding clinicians to prescribe appropriate antibiotics but also for evidence-based recommendations in empirical antibiotic treatment of wound and other infections [15, 16]. This evidence based prescribing is highly needed everywhere antibiotics are prescribed.

The aim of this study is to identify the microbial contaminants of wounds in patients admitted in a tertiary hospital in Nigeria, determine the incidence and antibiogram of ESBLs producing bacteria and point out the incidence of multi-drug resistant ESBLs producing isolates. This will help to provide evidence-based recommendations for empirical antibiotic treatment of infections caused by ESBL producing bacteria.

## Methods

### Study area

This is a prospective experimental study designed and carried out in Chukwuemeka Odumegwu Ojukwu University Teaching Hospital Amaku-Awka, a 150 bed space hospital. The hospital serves an urban community-Awka, the State capital and several rural communities with a total population of over 2 million persons. It is a state-owned tertiary health institution providing medicare to patient as well as medical and nursing training.

### Patients’ identification and sample collection

The patients were drawn from medical, surgical, obstetrics, pediatric and emergency (both children and adults) wards as well as the Intensive care unit of the hospital. Every patient in the wards with wound of at least one week duration (except in emergency wards which were two days duration) was recruited and wound swab samples were collected between March and June 2015 by a medical personnel using sterile swab-stick. Samples collected were analyzed within 30 minutes of collection. Verbal informed consent was gotten from the patients or their legal relatives after due explanation of the study protocols and purpose.

### Isolation and identification of organisms

The swab sticks used for the collection of the samples were streaked directly on Mac-conkey, Mannitol salt, Cetrimide and Chocolate blood agars and incubated at 37^o^C for 24 hrs. After incubation, cultures were examined for significant growth. Subcultures were plated onto nutrient agar plates and incubated for another 24 hrs. Grams staining and biochemical tests (Indole production, Oxidase, catalase and Coagulase) were carried out in accordance with standard methods [17].

### Preliminary screening of isolates

Gram staining reaction was carried out to distinguish the Gram positive and Gram negative isolates. Briefly, a minute quantity of the isolates colonies was aseptically transferred from the Petri dish on to a sterile slide previously wetted with a few loopful of water. The culture was spread evenly and gently with an inoculation loop to make a small circular thin film and then air-dried and fixed over a gentle flame. Crystal violet stain was added over the fixed culture and allowed to stand for 10-60 seconds. Enough mordant (iodine solution) was added to cover the fixed culture and allowed to stand for another 10-60 seconds. The slide was rinsed with running water, thereafter, carefully cleansed to wipe off the excess water from its surface. A few drops of alcohol solution (a decolorizer) were added such that the solution trickled down the slide. After optimal decolonization, the slide was again rinsed with water and basic fuchsin solution (a counterstain) was applied for 40-60 seconds. After washing with water, the slide was air-dried and then viewed under the microscope.

### Sugar fermentation test

The Mannitol Fermentation Test is a general test that confirms all the *Enterobacteriaceae* isolates. They produce gas on metabolizing mannitol. Briefly, an inoculum from the pure culture was aseptically transferred to a sterile tube containing phenol red mannitol broth (nutrient broth to which 0.5-1.0% mannitol was added) and incubated at 35-37^0^C for 18-24 hrs. Mannitol fermenters *(Enterobacteriaceae)* changed the colour of the broth from red to yellow, indicating acid-gas production.

The lactose fermentation is a general test that differentiates lactose fermenting *Enterobacteriaceae* e.g. *Escherichia coli* and *Klebsiella spp* from non-lactose fermenters e.g. *Salmonella spp and Shigella spp*. The procedure is similar to that of mannitol fermentation test except that phenol red lactose broth (nutrient broth to which 0.5-1.0% lactose was added) instead of phenol red mannitol broth was used. Lactose fermenters turned the broth yellow instead of red indicating acid production.

### Biochemical tests

**Catalase test:** The Gram positive isolates, each, was smeared on to a sterile slide. With a sterile wire loop few drops of hydrogen peroxide was added to the smear on a microscope slide and emulsified. This test differentiates catalase producing e.g. *Staphylococcus aureus* from non-catalase producing bacteria e.g. *Streptococci*.

**Coagulase test:** Distinguishes pathogenic *S. aureus* (positive test) from non-pathogenic *Staphylococcus epidermidis* (negative test). A clean microscope glass slide was divided into two sections (A and B) using a grease pencil. Section A was “test” and Section B “control”. A small drop of normal saline was placed on each section and then a small amount of the isolates was picked from the Petri plate using sterilized cooled inoculating loop and emulsified on section A to make a smooth smear. A drop of hydrogen peroxide was placed over the test smear. Nothing was put in the other drop/section B that served as control. Positive tests showed an evolution of gas bubbles.

**Oxidase test:** This test was carried out with all the Gram negative isolates to distinguish *Pseudomonas aeruginosa* from other enteric bacteria. Two drops of oxidase reagent was placed on a filter paper on a slide, a colony of the test organism was collected using a previously flamed and cooled wire loop, and smeared across the emulsified filter paper.

**Indole test:** The indole reagents was prepared as follows; A 3ml of sterile tryptone water was placed in a bijou bottle, after which the isolates were inoculated in these bottles and incubated at 35-37°C for up to 48 hrs. Indole ring formation was tested by adding 0.5ml of Kovac’s reagent with a gentle shake.

### Screening for ESBL bacteria

This was done using Double Disc Synergy Test (DDST) [18]. Briefly, 20 ml of Mueller Hinton agar was prepared and dispensed aseptically into a Petri dish and was allowed to solidify. The plates were seeded with test organisms pre-adjusted to 0.5 McFarland turbidity standards. A combination disc of co-amoxiclav (amoxicillin 20 μg and clavulanic acid 10 μg) was then placed at the center of the Petri dish and between two cephalosporin antibiotics (ceftazidime 30 μg and ceftriaxone 30 μg) placed 30 mm apart. The set up was done in duplicate and allowed for 30 minutes for pre-diffusion. It was incubated at 37°C for 18-24 hrs after which the various Inhibition Zone Diameters (IZDs) were measured. Enhanced zone of inhibition between any of the beta-lactam discs and the centre disc was recorded. In this study, an enhanced zone of inhibition between any of the third generation cephalosporin antibiotic discs and the co-amoxiclav disc was confirmation of ESBL production [19].

### Antibiotic susceptibility of the ESBL bacteria isolates

This was done using disc diffusion method. Briefly, 20 ml Mueller Hinton agar was prepared and dispensed aseptically into a Petri dish and was allowed to solidify. Standardized 0.5 McFarland inoculum size (10^6^ CFU/mL) of test bacteria was seeded on to the Mueller Hilton agar. The antibiotic disc was aseptically placed on the surface of the Mueller-Hinton agar and was allowed for 30 minutes for pre-diffusion. The set up was done in triplicate with a control containing no antibiotics disc. They were incubated for 24 hrs at 37°C after which the inhibition zone diameters were measured. The antibiotics used were: Azithromycin (15 mg), Erythromycin (30 mg), Ciprofloxacin (5 mg), Sparfloxacin (15mg), gentamicin (10 mg), Streptomycin Ceftriaxone (30 mg), Cefuroxime (30 mg), Ampicillin (10 mg), and amoxicillin/clavulanic acid (20/10 mg). Susceptibility/Resistance was interpreted using Clinical and Laboratory Standard Institute (CLSI) guidelines [19].

**Method of data analysis:** Data was analyzed using simple percentage.

### Ethical approval and compliance with ethical standards

The ethical approval was obtained from the Ethical Committee of the Hospital Management (Approval Number: HD92/A2/37). The study was conducted following the international, national, and institutional ethical guidelines and according to the 1964 Helsinki declaration and its later amendments or comparable ethical standards. The participants gave verbal consent, to collect the wound swabs, after the purpose of the study and intentions to publish the findings were explained. Patients were assured that their personal identities will not be linked to any data.

## Results

The wounds evaluated were diabetic sore (14), cancer wounds (12), surgical wounds (17), wounds due to road traffic accidents (10) and wounds from fire burn (10)-totaling 63. The result showed that 61 wounds were infected with microorganism. Fifty (50) isolates were Gram negative out of which 22 were negative in the mannitol sugar fermentation test (Table 1). The twenty-two (36.07%) isolates were oxidase test positive confirming *Pseudomonas aeruginosa*. The present study showed that *P. aeruginosa* was the leading pathogen (an ESBL producer) in the hospital wound with a prevalence of 36.07%. Eleven (11) isolates were Gram positive, catalase and coagulase positive, fermented mannitol sugar, and were confirmed *Staphylococcus aureus*. Thus, *S. aureus* has 18.03% incidence among the isolates. Eleven (11) of the Gram negative isolates fermented mannitol and lactose and were indole test positive, confirming *E. coli*. Nine Gram negative isolates fermented mannitol and lactose and were urea test positive but indole test negative confirming *Klebsiella pneumonia*. The incidence rate of E. coli was 3.28% in post-operative wound infections (Table 1). Eight isolates were Gram-negative, motile, short rods but oxidase-, urease- and indole-negative and fermented triple sugar iron agar (0.1% glucose + 1.0% lactose + 1.0% sucrose + 0.02% ferrous sulfate + phenol red + nutrient agar) with copious H_2_S gas production (giving black precipitate) confirming *Salmonella typhi*.

**Table 1:** Isolates from different wound sources

Source (wounds)	No Isolates	Escherichia coli	Pseudomonas aeruginosa	Klebsiella pneumoniae	Salmonella typhi	Staphylococcus aureus	Total
Surgery	2 (3.17%)	2 (3.17%)	5 (7.94%)	3 (4.76%)	1 (1.59%)	4 (6.35%)	17 (26.98%)
Accident^+^	0 (0.00%)	1 (1.59%)	10 (15.87%)	3 (4.76%)	4 (6.35%)	2 (3.17%)	20 (31.75%)
Cancer	0 (0.00%)	2 (3.17%)	4 (6.35%)	1 (1.59%)	3 (4.76%)	2 (3.17%)	12 (19.05%)
Diabetes	0 (0.00%)	6 (9.52%)	3 (4.76%)	2 (3.17%)	0 (0.00%)	3 (4.76%)	14 (22.22%)
Total	2 (3.17%)	11 (17.46%)	22 (34.92%)	9 (14.28%)	8 (12.79%)	11 (17.46%)	63 (100 %)

+ Accidents comprised Road Traffic Accident and Fire Burn

ESBL production is positive when there is expansion of inhibition zone of the combination disc to other single cephalosporin disc. The present study (Table 2) showed that slightly above 50% of the isolates were ESBL producers. Among the 22 *Pseudomonas aeruginosa* isolates, 18 (81.82%) produced ESBL. None of the *Staphylococcus aureus* isolates produced the β-lactamase enzyme while only one of the *Salmonella typhi* isolates produced the enzyme. Also, in this study, 20 (32.79%) lactose fermenters were recorded with ESBL production detected in 90.91% of *E. coli* isolates and 55.56% of *Klebsiella* isolates.

**Table 2:** ESBL confirmation test using double disc synergy tests method

Source (wounds)	*Escherichia coli* n (positive)	*Pseudomonas aeruginosa* n (positive)	*Klebsiella pneumoniae* n (positive)	*Salmonella typhi* n (positive)	*Staphylococcus aureus* n (positive)	Total n (positive)
Surgery	2 (2)	5 (3)	3 (2)	1 (0)	4 (0)	15 (7)
Accidents^+^	1 (1)	10 (10)	3 (2)	4 (1)	2 (0)	20 (10)
Cancer	2 (1)	4 (3)	1 (0)	3 (0)	2 (0)	12 (4)
Diabetes	6 (6)	3 (2)	2 (1)	0 (0)	3 (0)	14 (9)
Total	11 (10)	22 (18)	9 (5)	8 (1)	11 (0)	61 (34)

+ Accidents comprised Road Traffic Accident and Fire Burn Non-ESBL producers = 27, n = number of isolates tested, (positive) = number of isolates positive for ESBL production

The result showed that the ESBL producing isolates were ampicillin (100%), ceftriaxone (61.76%) and cefuroxime (94.12%) resistant respectively (Table 3, Table 4). The isolates expressed resistance of 32.35% and 47.06%, respectively to the quinolones (sparfloxacin and ciprofloxacin). The isolates also exhibited some resistance to the aminoglycosides (gentamicin, streptomycin) being 23.53% resistant to gentamicin and 58.82% resistant to streptomycin. They were all resistant to ampicillin and none were resistant neither to amoxicillin/clavulanate nor to ceftriaxone/sulbactam. The macrolides, azithromycin and erythromycin, has 35.29% and 41.18% microbial resistance respectively. In the present study, the resistance rates of ESBL producing isolates to ceftriaxone and cefuroxime were found to be 61.76% and 94.12% respectively in the infected wounds of the hospitalized patients. *Pseudomonas aeruginosa* (the most prevalent isolate in the present study) was 66.67% and 100% resistant to the two cephalosporins. The wound ESBL producing isolates were 61.76% and 94.12% resistant to the tested cephalosporins.

**Table 3:** Susceptibility patterns of the ESBL isolates

S/N	Susceptibility pattern of the ESBL *Pseudomonas aeruginosa* Isolates (n = 18)																		
	Antibiotics	Inhibition Zone Diameter (IZD in mm)																	
		*1*	*2*	*3*	*4*	*5*	*6*	*7*	*8*	*9*	*10*	*11*	*12*	*13*	*14*	*15*	*16*	*17*	*18*
1	Azithromycin	23	12	30	24	25	31	30	8	29	23	13	13	30	24	15	26	27	8
2	Erythromycin	23	25	23	23	9	12	0	0	11	25	23	12	13	14	15	23	7	8
3	Ciprofloxacin	0	23	3	4	24	23	23	8	23	23	11	12	13	25	25	11	27	23
4	Cefuroxime^+^	10	0	5	0	11	12	11	9	10	10	10	11	11	4	5	13	12	8
5	Ceftriaxone^+^	0	2	3	0	23	6	7	23	23	23	11	12	24	24	10	6	7	8
6	Streptomycin^+^	23	2	3	4	25	24	7	23	23	10	11	24	23	14	5	24	10	23
7	Gentamicin	0	23	23	24	23	23	7	24	23	23	24	12	23	27	23	23	25	23
8	Sparfloxacin	23	2	23	23	5	6	24	8	9	24	23	23	23	24	26	26	27	23
9	Ampicillin^+^	0	0	0	0	0	0	0	0	0	10	0	2	3	9	6	6	10	0
10	Co-amoxiclav	31	32	26	30	31	31	25	26	25	30	31	32	27	25	24	26	25	25
11	Ceftriaxone/sulbactam	26	28	30	32	30	26	27	30	30	20	31	26	28	31	27	28	25	25
	**Susceptibility pattern of the ESBL *Escherichia coli* (n = 10)*, Klebsiella pneumoniae* (n = 5) and *Salmonella typhi* (n = 1)Isolates**																	
		***Escherichia coli***											***K. pneumoniae***						***S. typhi***
1	Azithromycin	0	24	23	23	24	25	0	28	23	23		10	12	0	24	5		30
2	Erythromycin	23	24	23	25	26	26	24	8	25	25		10	12	30	24	25		23
3	Ciprofloxacin	10	24	0	4	5	24	25	28	9	13		23	23	10	24	5		24
4	Cefuroxime^+^	10	12	23	4	5	11	10	8	23	12		11	12	0	4	12		9
5	Ceftriaxone^+^	9	10	23	24	12	7	26	0	23	25		25	24	0	4	5		8
6	Streptomycin^+^	13	10	0	4	25	24	0	8	25	13		26	12	0	11	25		4
7	Gentamicin	23	23	23	24	25	31	25	28	9	23		10	10	25	23	24		24
8	Sparfloxacin	23	12	0	24	24	10	26	28	12	24		25	23	25	4	6		30
9	Ampicillin^+^	10	12	0	4	5	10	0	8	9	3		0	2	0	8	5		0
10	Co-amoxiclav	30	26	30	24	25	24	30	28	29	23		23	23	24	26	30		28
11	Ceftriaxone/sulbactam	30	32	30	34	25	31	30	29	24	28		32	30	26	28	31		28

**Table 4:** Individual ESBL producing isolates’ resistance to conventional antibiotics

S/N	Antibiotics	Number of the isolates resistance to commercial antibiotics (%)				
		*P. aeruginosa* N = 18	*E. coli* N = 10	*K. pneumonia* N = 5	*S. typhi* N = 1	Total N = 34
1	Azithromycin	6 (33.33)	2 (20)	4 (80)	0	12 (35.29)
2	Erythromycin	11 (61.11)	1 (10)	2 (40)	0	14 (41.18)
3	Ciprofloxacin	8 (44.44)	6 (60)	2 (40)	0	16 (47.06)
4	Cefuroxime^+^	18 (100)	8 (80)	5 (100)	1 (100)	32 (94.12)
5	Ceftriaxone^+^	12 (66.67)	5 (50)	3 (60)	1 (100)	21 (61.76)
6	Streptomycin^+^	9 (50.00)	7 (70)	3 (60)	1 (100)	20 (58.82)
7	Gentamicin	3 (16.67)	3 (30)	2 (40)	0	8 (23.53)
8	Sparfloxacin	5 (27.78)	4 (40)	2 (40)	0	11 (32.35)
9	Ampicillin^+^	18 (100)	10 (100)	5 (100)	1 (100)	34 (100)
10	Co-amoxiclav	0	0	0	0	0
11	Ceftriaxone/sulbactam	0	0	0	0	0

+ Greater than 50% of the ESBL producing isolates were resistant

Multi-Antibiotics Resistance Index (MARI) analysis of the ESBL producing Isolates (Table 5) revealed that the minimum number of antibiotics to which the isolates were resistant was 3 and the maximum was 8 out of the 11 antibiotics used. All the isolates had a MARI of greater than 20% giving an incidence of Multi-Antibiotics Resistance strains of 100%.

**Table 5:** Multi-Antibiotics Resistance Index (MARI) analysis of the ESBL producing Isolates

S/N	ESBL Isolates	Number of Isolates	Number of isolates resistant to ≥3 antibiotics	Number of antibiotics to which the isolates were resistant (Range)	Average number of antibiotics to which the isolates were resistant (a)	MARI (a/b) in %
1	*P. aeruginosa*	18	18	3-8	4.72	42.91
2	*E. coli*	10	10	3-7	4.70	42.73
3	*K. pneumoniae*	5	5	5-7	5.40	49.09
4	*S. typhi*	1	1	4	4.00	36.36
5	Total	34	34	3-8		

Antibiotics used: azithromycin, erythromycin, ciprofloxacin, sparfloxacin, gentamicin, streptomycin, ceftriaxone, cefuroxime, ampicillin, ceftriaxone/sulbactam and co-amoxiclav

## Discussion

This result (Table 1) showed that the rate of isolation of Gram-negative organisms was more than Gram-positive and is consistent with several other reports [10, 20–22]. The reason why *P. aeruginosa* was the most isolated pathogen in this study could be because it is a common cause of nosocomial infection [21, 22] as well an opportunistic pathogen that thrives more wherever there is suppressed immunity as seen in wound infections. The incidence rate of the wounds infections was high (96.83%) probably due to factors associated with the acquisition of nosocomial pathogens in patients with recurrent or long-term hospitalization, complicating illnesses, prior administration of antimicrobial agents, or the immunosuppressive effects of wound trauma. According to Iroha et al., [11], β-lactam antibiotics are the most frequently prescribed antibiotics against aerobic Gram negative bacilli infections in Nigeria, and selective pressure exerted by the extensive use of these β-lactam drugs, most likely resulted in strains developing ESBL enzymes. Occurrence and distribution of ESBLs differ from country to country and from hospital to hospital.

The present study (Table 2) also showed that the ESBL producers were coming mainly from the non-*Enterobacteriaceae* family-*Pseudomonas* genera (36.07%). Again, this takes the advantage of suppressed immunity caused by wound trauma. This result differs from the reports by Afunwa *et al.,* [12] and Ankur *et al.,* [23]. They reported that the *Enterobacteriaceae* family is the major ESBL producer. The cause of the discrepancy could be because most of their samples came from immunocompetent patients. They also identified the multi-drug resistance (MDR) bacteria strains to belong principally to the *Enterobacteriaceae* family and *Pseudomonas* genera with a greater percentage skewing towards the *Enterobacteriaceae* category. Mathur *et al.,* [24] documented the prevalence of ESBL producers to be as high as 68.0%, whereas Kumar *et al.,* [25] reported 19.2% of E. coli isolates as ESBL producers. This is contrary to 20.27 % ESBL producing isolates of *P. aeruginosa* reported by Aggarwal *et al.,* [26] proving the increasing incidence of the organism in hospitalized patients [27, 28].

*Salmonella enteric* Typhi serovar is a Gram-negative flagellated short rod of the *Enterobacteriaceae* family. The possible contamination of the wounds by this organism in our study revealed possible spread from environment, contaminated water and/or undercooked food. One of the wounds was contaminated with ESBL producing *Salmonella enterica* Typhi serovar. Previous reports confirmed the occurrence of this organism and showed that multi-drug resistance strains are emerging [2, 29, 30]. This shows the emergence of plasmid mediated ESBLs among members of *Enterobacteriaceae* in the hospital.

The high rate of microbial resistance to the quinolones (47.06% and 32.35%) as observed in this study (Table 3, Table 4) is a cause for concern as many clinicians fall back on them for the treatment of Gram-negative pathogens in the face of multi-drug resistance [31]. The high incidence of the isolates resistant to the quinolones may be attributed to cross-resistance to the class occasioned by high non-prescription use of ciprofloxacin in the area as well as possible drug faking. The results of the antibiotic resistance profile of the isolates to beta-lactam antibiotics showed increased beta-lactamase enzyme production among the isolates. Many of the ESBL producing isolates were resistant to ceftriaxone (61.76%) and cefuroxime (94.12%) respectively. A similar observation was made by [32] who also reported the isolation of cephalosporin resistant *Pseudomonas aeruginosa* among in-patients and out-patient clinics. They explained that the duration of the hospital stay was directly proportional to a higher prevalence of the infection. Aminoglycosides (streptomycin and gentamicin), on the average, had less activity and showed more microbial resistance profile compared to macrolides (Table 4). The reason for this high resistance to the aminoglycosides could be as a result of indiscriminate use of streptomycin in the area. The drug though a prescription only medicine is purchased as over-the-counter in the open markets littered in the community and is commonly used by unqualified personnel in the treatment of “infections”. Several studies show that gentamicin and streptomycin are effective against *Pseudomonas* species and *Escherichia coli*, but if misused, the organisms may also develop resistance to them [32, 33]. Our findings are similar to that of Sasirekha *et al.,* [33], where they recorded 82.1% susceptibility of the isolates to gentamicin and 41.8% to streptomycin probably although they worked on clinical isolates generally and not on ESBL producers alone. The high resistance profile of the isolates in this study is a reflection of the high incidence of ESBL isolates observed. *Pseudomonas aeruginosa*, much like *Mycobacterium tuberculosis* is intrinsically resistant to several antimicrobial classes and therefore poses considerable limitation in the range of antibiotic options for treating infections caused by them. These increase further the risk for emergence of resistant strains. Microorganisms usually encode multiple antibiotic resistance genes [34].

Many of the ESBL producing isolates (61.76% and 94.12%) were resistant to the cephalosporins used (ceftriaxone and cefuroxime respectively) in the study (Table 3). This is contrary to the study done by Sasirekha *et al.,* [33] in India where they found resistance rates to be as few as 24% and 29. May be they did not encounter ESBL producers in their study. It was also noted that *Pseudomonas* and *Klebsiella* species were more resistant than *Escherichia coli*. This may be due to their inherent virulence factors like hyper-viscosity, polysaccharide capsules and production of endotoxin [35].

Multi-Antibiotics Resistance results when microbes are resistance to tested antibiotics in at least two of the classes: quinolones, aminoglycosides and β-lactams [36, 37]. It is identified by profiling the pattern of resistance of microbial isolates to the antibiotics belonging to the above classes.

The multi-antibiotic resistance index is calculated by the formula: a/bx100. a = Average number of antibiotics to which the isolates were resistant = Sum of the number of antibiotics to which the isolates were resistant/Number of resistant isolates b = Total number of antibiotics the isolates were subjected to = 11 in this study

The incidence of multi-antibiotic resistant isolates was calculated as: 

$$\frac{\text{Number}\;\text{of}\;\text{isolates}\;\text{with}\;\text{MARI}>20\%}{\text{Total}\;\text{Number}\;\text{of}\;\text{Isolates}}\times 100$$

=34/34x100 = 100 %

All the ESBL producing isolates were resistant to multiple antibiotics (Table 5). The incidence of Multi-Antibiotics Resistance strains is extremely high in the hospital community suggesting poor antibiotic stewardship such as lack of antibiotic policy, irrational use/prescribing of antibiotics and the emergence of antibiotic-resistant strains in the hospital. Antibiotic misuse is a common occurrence in developing countries due to chaotic drug distribution and existence of open-drug markets in the area [38, 39]. Antibiotics abuse and misuse and/or non-compliance to prescribed antibiotics are some of the factors encouraging the spread of multi-drug resistant bacteria.

## Conclusion

The incidence of Multi-Antibiotics Resistant strains is extremely high in the hospital community. Amoxicillin/clavulanate or ceftriaxone/sulbactam is recommended for empirical treatment of infections caused by ESBL-producing bacteria in the hospital. This may guide antibiotic selection and use in trauma, most especially in resource limited countries where laboratory test is unaffordable for a majority of patients.

### What is known about this topic

- Antibiotic resistance is a concern to both the clinicians and their patients;
- Extended spectrum β-lactamase (ESBLs) producing organisms are implicated in hospital-acquired infections;
- Antibiotic selection for infections due to ESBL-producing pathogens is a clinical challenge.

### What this study adds

- High incidence (55.74 %) of ESBL-producing microbes in the wound and skin infections, non-enterobacteriaceae being the dominating isolates;
- The multi-antibiotic resistance index calculation showed that the incidence of multi-antibiotic resistance in the study was 100%;
- The emergence of ESBL-producing *Salmonella spp*.

## Competing interests

Authors wish to declare that the author, Ifeanyi Ezeobi is an employee of Chukwuemeka Odumegwu Ojukwu University Teaching Hospital Amaku-Awka, Anambra State and a native of the state. The author, Angus Nnamdi Oli is a native of Anambra State where the hospital is located. The authors, Dennis Emeka Eze, Thaddeus Harrison Gugu, Ukamaka Nwakaku Maduagwu and Chibueze Peter Ihekwereme declare no competing interest.

## References

[1] Aibinu IE, Ohagbulam VC, Adenipekun EA, Ogunsola FT, Odugbemi TO, Mee BJ (2003). Extended-spectrum beta-lactamase enzymes in clinical isolates of Enterobacter species from Lagos, Nigeria. J ClinMicrobiol.

[2] Bharat Pokharel M, Janak Koirala, Rajan Dahal K, Shyam Mishra K, Prem Khadga K, Tuladhar NR (2006). Multidrug-resistant and extended-spectrum beta-lactamase (ESBL)-producing *Salmonella enterica* (serotypes Typhi and Paratyphi A) from blood isolates in Nepal: surveillance of resistance and a search for newer alternatives. International Journal of Infectious Diseases.

[3] Maina Daniel, Makau Paul, Nyerere Andrew, Revathi Gunturu (2013). Antimicrobial resistance patterns in extended-spectrum β-lactamase producing *Escherichia coli* and *Klebsiella pneumoniae* isolates in a private tertiary hospital, Kenya. Microbiology Discovery.

[4] Fang Hong, Ataker Ferda, Hedin Go¨ran, Dornbusch Kathrine (2008). Molecular epidemiology of extended-spectrum β-lactamases among *Escherichia coli* isolates collected in a Swedish hospital and its associated health care facilities from 2001 to 2006. J Clin Microbiol.

[5] Oluduro Anthonia Olufunke, Aregbesola Oladipupo Abiodun, Fashina Christina Dunah (2014). 2014 Extended Spectrum Beta- Lactamase- Producing Uropathogenic *Escherichia coli* in Pregnant Women Diagnosed With Urinary Tract Infections in South-Western Nigeria. Journal of Molecular Biology Research.

[6] Paterson DL, Bonomo RA (2005). Extended-spectrum β-lactamases: a clinical update. Clin Microbiol Rev.

[7] Ramphal R, Ambrose PG (2006). Extended-spectrum beta-lactamases and clinical outcomes: current data. Clin Infect Dis.

[8] Pitout Johann DD, Nordmann Patrice, Laupland Kevin B, Poirel Laurent (2005). Emergence of Enterobacteriaceae producing extended-spectrum β-lactamases (ESBLs) in the community. J Antimicrob Chemother.

[9] Arzai AH, Adamu DJM (2008). Prevalence of beta-lactamase Producers among randomly selected bacterial pathogens in Kano, Nigeria. Biological and Environmental Sciences Journal for the Tropics.

[10] Yusuf I, Haruna M, Yahaya H (2013). Prevalence and antibiotic susceptibility of ampc and ESBL producing clinical isolates at a tertiary health care center in Kano, northwest Nigeria. African Journal of Clinical and Experimental Microbiology.

[11] Iroha IR, Adikwu MU, Esimone CO, Aibinu I, Amadi ES (2009). Extended spectrum Beta-Lactamase (EBSL) in *E coli* isolated from a tertiary hospital in Enugu state, Nigeria. Pak J Med Sci.

[12] Afunwa RA, Odimegwu DC, Iroha RI, Esimone CO (2011). Antimicrobial resistance status and prevalence rates of extended spectrum beta-lactamase (ESBL) producers isolated from a mixed human population. Bosn J Basic Med Sci.

[13] Song W, Lee KM, Kang HJ, Shin DH, Kim DK (2001). Microbiologic aspects of predominant bacteria isolated from the burn patients in Korea. Burns.

[14] Oli Angus Nnamdi, Ekejindu Callistus Chibuike, Ejiofor Obiora Shedrack, Oli Adaobi Helen, Ezeobi Ifeanyi, Ibeh Christian Chibuzo (2016). The knowledge of and attitude to hospital-acquired infections among public and private healthcare workers in South-East, Nigeria. British Journal of Medicine & Medical Research.

[15] Blomberg B, Jureen R, Manji KP, Tamim BS, Mwakagile DS, Urassa WK, Fataki M, Msangi V, Tellevik MG, Maselle SY, Langeland N (2005). High rate of fatal pediatric septicemia caused by gram-negative bacteria with extended-spectrum beta lactamases in Dares Salaam, Tanzania. Journal of Clinical Microbiology.

[16] Leekha Surbhi, Terrell Christine L, Edson Randall S (2011). General principles of antimicrobial therapy. Mayo Clin Proc.

[17] Cheesbrough Monica (2006). District Laboratory Practice in Tropical Countries Part 2.

[18] Jarlier V, Nicolas MH, Fournier G, Philippon A (1998). Extended broad spectrum β-lactamases conferring transferable resistance to new β-lactam agents in Enterobacteriaceae Hospital prevalence and susceptibility patterns. Rev Infect Dis.

[19] Clinical and Laboratory Standards Institute (CLSI) Performance Standards for Antimicrobial Disk Susceptibility Tests; Approved Standard-Eleventh Edition (2012). CLSI document M02-A11 (ISBN 1-56238-781-2 [Print]; ISBN 1-56238-782-0 [Electronic]). Clinical and Laboratory Standards Institute.

[20] Kehinde AO, Ademola SA, Okesola AO, Oluwatosin OM (2004). Pattern of bacterial pathogens in burn wound infections in Ibadan, Nigeria. Annals of Burns and fire disasters.

[21] Mehedi HM, Arongozeb G, Muktadir K, Zakaria A (2013). Isolation and identification of different bacteria from different types of burn wound infections and study their antimicrobial Sensitivity pattern-IMPACT. International Journal of Research in Applied, Natural and Social Sciences.

[22] Manjula M, Priya D, Varsha G (2007). Bacterial isolates from burn wound infections and antibiograms: eight year study. Ind J Plastic Surg.

[23] Ankur Goyal, Prasad KN, Amit Prasad, Sapna Gupta, Ujjala Ghoshal, Archana Ayyagari (2009). Extended spectrum β-lactamases in *Escherichia coli* and *Klebsiella pneumoniae* & associated risk factors. Indian J Med Res.

[24] Mathur P, Tatman A, Das B, Dhavan B (2002). Prevalence of ESBL gram negative bacteria in a tertiary care hospital. Indian J Med Microbiol.

[25] Kumar MS, Lakshmi V, Rajagopalan R (2006). Occurrence of extended spectrum beta lactamases among *Enterobacteriaceae* spp isolated at a tertiary care institute. Indian J Med Microbiol.

[26] Aggarwal R, Chaudhary U, Bala K (2008). Detection of extended spectrum β- lactamase in *Pseudomonas aeruginosa*. Indian J Pathol Microbial.

[27] Alharbi Sulaiman A, Zayed ME (2014). Antibacterial susceptibility of bacteria isolated from burns and wounds of cancer patients. Journal of Saudi Chemical Society.

[28] Shaikh Sibhghatulla, Fatima Jamale, Shakil Shazi, Mohd Syed, Rizvi Danish, Kamal Mohammad Amjad (2015). Prevalence of multidrug resistant and extended spectrum beta-lactamase producing *Pseudomonas aeruginosa* in a tertiary care hospital. Saudia Journal of Biological Sciences.

[29] González-López Juan José, Piedra-Carrasco Nuria, Salvador Fernando, Rodríguez Virginia, Sánchez-Montalvá Adrián, Planes Anna M, Israel Molina, Larrosa M (2014). Nieves; ESBL-Producing *Salmonella enterica* Serovar Typhi in Traveler Returning from Guatemala to Spain. Emerging Infectious Diseases.

[30] Gautam K, Pokhre BM, Bhatta DR, Shrestha CD (2012). Studies on Extended Spectrum Beta Lactamase (ESBL) producing *Salmonella* isolates from clinical samples of Nepal. Nepal Med Coll J.

[31] Paterson DL, Doi Y (2007). A step closer to extreme drug resistance (XDR) in gram-negative bacilli. Clin Infect Dis.

[32] Anupurba S, Battacharjee A, Garg A, Ranjansen M (2006). The antimicrobial Susceptibility of *Pseudomonas aeruginosa* isolated from wound infections. Indian J Dermatol.

[33] Sasirekha B, Monasa R, Ramya P, Sneha R (2010). Frequency and Antimicrobial sensitivity Pattern of Extended Spectrum B-Lactamases Producing *Escherichia coli* and *Klebsiella pneumonia* Isolated in A Tertiary Care Hospital, Al Ameen. Journal of Medical Science.

[34] Gad GF, Mohamed HA, Ashour HM (2011). Aminoglycoside Resistance Rates, Phenotypes, and Mechanisms of Gram-Negative Bacteria from Infected Patients in Upper Egypt. PLoS ONE.

[35] Perez F, Endimiani A, Hujer KM, Bonomo RA (2007). The continuing challenges of ESBLs. Curr Opin Pharmacol.

[36] Lin Y, Lu M, Hui-Ling Tang, Liu H, Chen H, Liu C, Lin K, Chiou C, Chiang C, Chen M, Yi-Chyi C, Lai Y (2011). Assessment of hypermucoviscosity as a virulence factor for experimental *Klebsiella pneumoniae* infections: comparative virulence analysis with hypermucoviscosity-negative strain. BMC Microbiology.

[37] Hill Dominic, Barbara Rose, Aniko Pajkos, Michael Robinson, Peter Bye, Scott Bell, Mark Elkins, Barbara Thompson, Colin MacLeod, Shawn Aaron D, Colin Harbour (2005). Antibiotic Susceptibilities of *Pseudomonas aeruginosa* Isolates Derived from Patients with Cystic Fibrosis under aerobic anaerobic and biofilm Conditions. J Clin Microbiol.

[38] Subramani S, Vignesh S (2012). MAR Index Study and MDR Character Analysis of a few Golden *Staph* Isolates. Asian J Pharmacy and Life Science.

[39] Adegoke AA, Komolafe AO (2009). Multi-drug resistant *Staphylococcus aureus* in clinical cases in Ile-Ife, Southwest Nigeria. Int J Med Sci.

